# Complement C3 activation regulates the production of tRNA-derived fragments Gly-tRFs and promotes alcohol-induced liver injury and steatosis

**DOI:** 10.1038/s41422-019-0175-2

**Published:** 2019-05-10

**Authors:** Fudi Zhong, Zhigao Hu, Keqing Jiang, Biao Lei, Zhan Wu, Guandou Yuan, Hongliang Luo, Chunqiang Dong, Bo Tang, Chaowen Zheng, Shuai Yang, Yonglian Zeng, Zhenya Guo, Shuiping Yu, Huizhao Su, Guo Zhang, Xiaoqiang Qiu, Stephen Tomlinson, Songqing He

**Affiliations:** 1 Division of Hepatobiliary Surgery, the First Affiliated Hospital of Guangxi Medical University, Nanning, 530021 Guangxi China; 2 Laboratory of Liver Injury and Repair, Nanning, Guangxi China; 3 Department of Gastroenterology, the People’s Hospital of Guangxi Zhuang Autonomous Region, Nanning, Guangxi China; 4 Department of Microbiology and Immunology, Medical University of South Carolina, Charleston, SC USA

**Keywords:** Innate immunity, Small RNAs

## Abstract

Complement is known to play a role in alcoholic fatty liver disease (AFLD), but the underlying mechanisms are poorly understood, thereby constraining the development of a rational approach for therapeutic intervention in the complement system. C3 deficiency has been shown to impart protective effects against ethanol-induced hepatic steatosis and inflammation. Here we demonstrate a protection effect in wild-type mice by treatment with CR2-Crry, a specific inhibitor of C3 activation. The expression of glycine transfer (t) RNA-derived fragments (Gly-tRFs) is upregulated in ethanol-fed mice and inhibition of Gly-tRFs in vivo decreases chronic ethanol feeding-induced hepatosteatosis without affecting inflammation. The expression of Gly-tRF was downregulated in C3-deficient or CR2-Crry-treated mice, but not in C5-deficient mice; Gly-tRF expression was restored by the C3 activation products C3a or Asp (C3a-des-Arg) via the regulation of CYP2E1. Transcriptome profiling of hepatic tissues showed that Gly-tRF inhibitors upregulate the expression of sirtuin1 (*Sirt1*) and subsequently affect downstream lipogenesis and β-oxidation pathways. Mechanistically, Gly-tRF interacts with AGO3 to downregulate *Sirt1* expression *via* sequence complementarity in the 3′ UTR. Notably, the expression levels of C3d, CYP2E1 and Gly-tRF are upregulated, whereas *Sirt1* is decreased in AFLD patients compared to healthy controls. Collectively, our findings suggest that C3 activation products contribute to hepatosteatosis by regulating the expression of Gly-tRF. Complement inhibition at the C3 activation step and treatment with Gly-tRF inhibitors may be potential and precise therapeutic approaches for AFLD.

## Introduction

Alcoholic liver disease is a leading cause of morbidity and mortality worldwide. Alcoholic fatty liver disease (AFLD) is a consequence of excessive alcohol consumption. When patients continue to drink, AFLD progresses to more severe forms of liver injury such as steatohepatitis, fibrosis, cirrhosis, and hepatocellular carcinoma.^1^ Excessive ethanol intake is estimated to be the cause of 4.5% of these diseases and 3.8% of all deaths around the world.^2,3^ AFLD is characterized by a number of symptoms, such as steatosis and inflammation, which contribute to liver injury. While AFLD has been extensively studied, current understanding of the pathogenesis of AFLD is still limited.

Several lines of evidence have shown that different mediators, such as the complement system, reactive oxygen species (ROS), neutrophils, macrophages and lipopolysaccharide (LPS), play critical roles in the development of AFLD. Complement activation is involved in ethanol-induced liver injury,^4,5^ and mice lacking complement component 3 (C3) are protected from ethanol-induced steatosis.^4^ In contrast, complement component 5 (C5) deficiency does not affect steatosis, although it imparts a protective effect on ethanol-induced inflammatory responses.^4^ Upon activation, C3 is cleaved into C3a and C3b, and C3b further promotes the activation/cleavage of C5 to C5a and C5b.^6^ C3a and C5a have been correlated with increased expression of cytokines and chemokines, which facilitate the progression of alcohol-induced liver inflammation. However, the underlying mechanisms of how C3 contributes to ethanol-induced steatosis are not well understood. Furthermore, there are no effective drugs for the therapy of AFLD. Liver transplantation is an accepted and successful therapy for severe AFLD patients, and liver resection is one of the radical treatments for hepatocellular carcinoma patients with an AFLD background. Liver ischemia-reperfusion injury (IRI) following surgery is a key contributor to liver dysfunction and failure, and there is currently no therapeutic regimen for preventing or minimizing hepatic IRI after transplantation or resection.

Complement receptor 2 (CR2)-Crry is a site-targeted complement inhibitor that blocks all complement pathways at the C3 activation step.^7^ The complement inhibitory moiety, Crry, is targeted via the CR2 fusion partner that binds C3d, a complement activation product deposited at sites of complement activation. We have previously shown that CR2-Crry can provide protection against hepatic IRI in some, but not all settings.^8,9^ Effective complement inhibtitory function of CR2-Crry is dependent on its localized targeting,^7^ and its effect on liver IRI in an AFLD background has not been previously investigated.

In the past two decades, various noncoding RNAs (ncRNAs) have been shown to play an important role in regulating gene expression. The development of small RNA high-throughput sequencing technologies has led to the discovery of diverse classes of ncRNAs. In addition to microRNAs (miRNAs), small RNAs can be processed from novel RNA sources such as transfer (t)RNA, small nucleolar (sno)RNA, and ribosomal (r)RNA.^10–13^ In recent years, small RNAs derived from tRNAs have been investigated in various research fields.^14–19^ Several lines of evidence indicate that tRNA-derived fragments (tRFs) are functional molecules involved in various biological processes,^14^^,^^20–22^ including the regulation of gene expression, and it has been shown that stress-induced tRFs can inhibit protein synthesis.^23^ Gebetsberger et al.^24^ showed that tRFs regulate translation during stress by competing with mRNA for ribosome binding. In addition, tRFs generated by specific cleavage of mature tRNAs are induced by a variety of stressors such as oxidative stress, heat/cold shock, and ultraviolet irradiation,^25–28^ and endonucleolytic cleavage of tRNAs is a widely conserved response to oxidative stress in eukaryotes.^29^ Moreover, angiogenin (ANG) is a stress-activated ribonuclease that is involved in tRF biogenesis.^23,27,28^ Ethanol consumption promotes the production of ROS, mainly hydrogen peroxide (H_2_O_2_) and superoxide anion (O_2_^−^), in the liver,^30^ and oxidative stress plays a prominent role in the pathogenesis of AFLD.^31–33^ Although the function of tRFs has been extensively studied, the role and molecular mechanisms of tRFs in AFLD are unknown.

SIRT1, an NAD^+^-dependent deacetylase, is a central molecule in hepatic lipid metabolism and AFLD pathogenesis.^34–37^ Deletion of the hepatic gene *Sirt1* results in stimulated lipogenesis and impaired β-oxidation,^38^ and SIRT1 triggers lipid metabolism by regulating numerous genes such as *Srebp-1c*,^34,39^ *Lipin1*,^38^ *Ppara*,^36^ and *Cpt1a*,^38^ which play major roles in AFLD development. Although a role for SIRT1 signaling in AFLD development is established, the molecular mechanisms associated with its disruption in response to ethanol challenge remain elusive.

In this study, we explored the underlying mechanisms whereby C3 contributes to ethanol-induced hepatic steatosis, and we assessed the therapeutic effect of CR2-Crry in ethanol-induced liver steatosis and inflammation in mice. Furthermore, we analyzed the roles of Gly-tRF and their link to complement activation in an AFLD mouse model, and evaluated potential therapeutic strategies using Gly-tRF antisense inhibitors.

## Results

### Therapeutic effect of targeted complement inhibitor CR2-Crry or C3 deficiency on ethanol-induced hepatic steatosis and liver injury

**Fig. 1:**
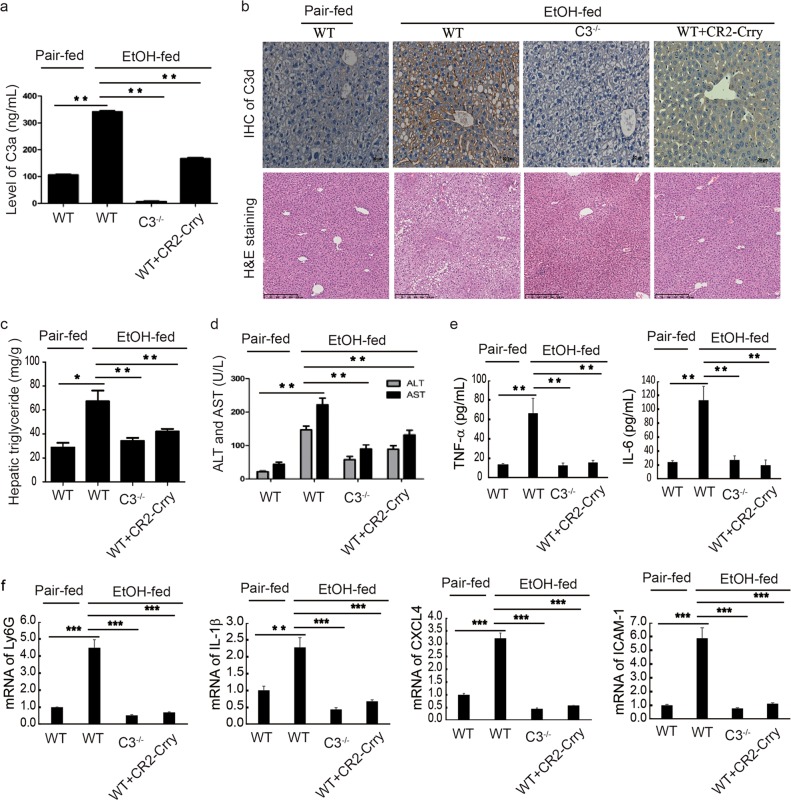
CR2-Crry treatment or C3 deficiency alleviates ethanol-induced steatosis and liver injury. Mice were subjected to short-term ethanol feeding. Ethanol-fed mice were injected i.p. with PBS or CR2-Crry (0.25 mg). There was no effect of PBS on WT mice; therefore, WT without treatment was used as controls instead of WT + PBS (omitted). a Serum levels of C3a in pair-fed, and ethanol-fed WT, *C3*^−/−^ and CR2-Crry-treated mice. **b** C3d deposition and H&E staining. **c** Hepatic triglyceride levels. **d** Serum ALT and AST levels in pair-fed, and ethanol-fed WT, *C3*^−/−^ and CR2-Crry-treated mice. **e** Serum levels of TNF-α and IL-6. **f** The mRNA levels of *Ly6G*, *IL-1β*, *CXC14* and *ICAM-1* were detected in short-term ethanol-fed mice. The data are representative of three independent experiments. Results are expressed as the means ± SD. *n* = 6–8, *P < 0.05; **P < 0.01,***P < 0.001

Studies have shown that C3 activation is involved in the development of AFLD.^4,5^ CR2-Crry is a site-targeted complement inhibitor of C3 activation,^7–9^ and humanized versions of site-targeted C3 inhibitors are in clinical development. We therefore investigated the therapeutic effect of CR2-Crry on ethanol-induced hepatic steatosis and inflammation. Mice were given ethanol *via* an intragastric gavage or by short-term binge feeding. Serum C3a levels were elevated after ethanol feeding in wild-type (WT) mice, but not in *C3*^−/−^ mice (Fig. 1a). C3a levels were decreased in ethanol-fed CR2-Crry-treated mice compared to untreated mice (Fig. 1a; Supplementary information, Fig. S1a). C3d accumulation also decreased in C3-deficient and CR2-Crry-treated mice compared to untreated mice after ethanol feeding (Fig. 1b; Supplementary information, Fig. S1b). Hematoxylin and eosin (H&E) staining showed that hepatic steatosis was significantly reduced in ethanol-fed CR2-Crry-treated mice compared to untreated mice, and fat deposits were almost undetectable in *C3*^−/−^ mice (Fig. 1b, c). Alanine aminotransferase (ALT) and aspartate aminotransferase (AST) levels were increased in the ethanol-fed WT mice compared to the pair-fed mice, but were significantly decreased in ethanol-fed CR2-Crry-treated WT mice as well as *C3*^−/−^ mice compared to untreated mice (Fig. 1d; Supplementary information, Fig. S1c). Inflammatory cytokines, such as tumor necrosis factor α (TNF-α) and interleukin-6 (IL-6) were upregulated in response to ethanol exposure, but C3 deficiency or CR2-Crry treatment imparted protective effects against ethanol-induced inflammation (Fig. 1e; Supplementary information, Fig. S1d, e). Furthermore, the expression levels of *Ly6G*, *IL-1β*, *Cxc14* and *ICAM1* were also elevated in ethanol-fed mice, but such elevation was abolished in *C3*^−/−^ or CR2-Crry-treated mice (Fig. 1f). Several lines of evidence indicate that ethanol metabolism impairs hepatocyte function, leading to apoptosis in AFLD.^40–42^ We therefore evaluated the effect of CR2-Crry on apoptosis. Hepatocyte apoptosis was markedly increased in ethanol-fed WT mice, but C3 deficiency or CR2-Crry treatment abolished the effect (Supplementary information, Fig. S2a). Apoptosis-related genes were further analyzed by western blot. We found that the expression levels of caspase-3, caspase-8, caspase-9 and Bax were elevated in the ethanol-fed mice, but this effect was reversed by C3 deficiency or CR2-Crry treatment. In contrast, Bcl2 expression was diminished in ethanol-fed mice, but was enhanced in C3-deficient or CR2-Crry-treated mice (Supplementary information, Fig. S2b). We next examined the expression of STAT3, p-STAT3, Akt and p-Akt in liver homogenates by western blot. The ratio of p-Akt/Akt expression was decreased in ethanol-fed mice, but C3 deficiency or CR2-Crry treatment increased the ratio compared to untreated mice. In addition, the ratio of p-STAT3/STAT3 expression was also increased by CR2-Crry treatment (Supplementary information, Fig. S2c).

### CR2-Crry treatment protects against IRI in AFLD mice

**Fig. 2:**
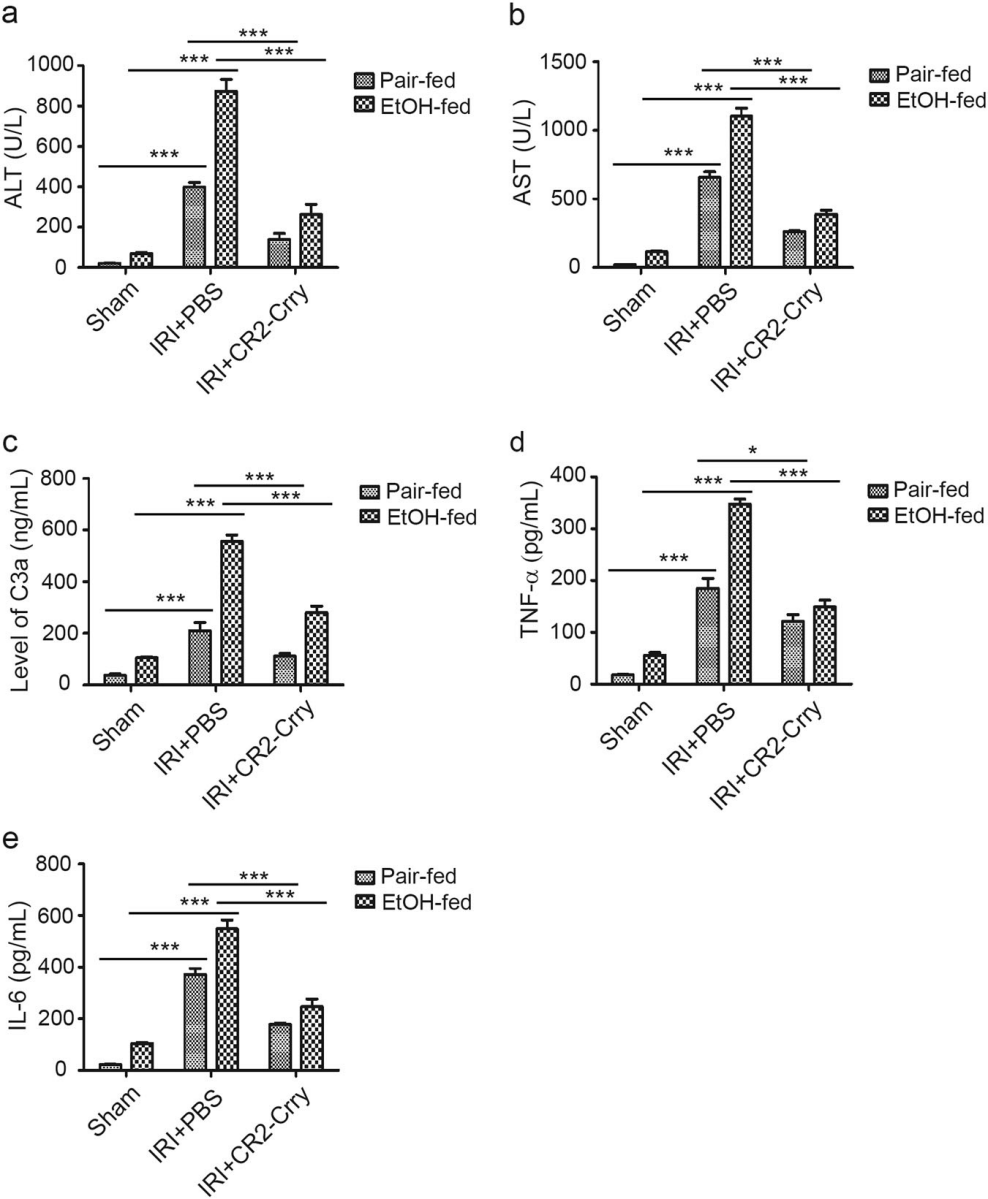
CR2-Crry treatment protects against liver IRI in AFLD mice. Liver IRI was performed in pair-fed or ethanol-fed mice treated with PBS or CR2-Crry. **a** Serum ALT levels. **b** Serum AST levels. **c** Serum C3a levels. **d** Serum TNF-α levels. **e** Serum IL-6 levels. The results are expressed as the mean ± SD. *P < 0.05, ***P < 0.001

Liver IRI is an unavoidable outcome in surgical procedures, and IRI is a major factor contributing to postsurgical liver dysfunction and liver failure.^8,43,44^ Complement activation can increase the susceptibility of steatotic livers to IRI.^44^ He et al.^8^ reported that CR2-Crry treatment protects against IRI in a murine ligation model, but not against liver IRI when ischemia is induced in the context of resection. It is therefore important to determine how targeted inhibition of C3 impacts liver IRI in an AFLD model. Mice were subjected to 30 min of total warm hepatic ischemia followed by reperfusion, and were treated with PBS or CR2-Crry 30 min before ischemia. After 6 h of reperfusion, we found that ALT and AST levels were elevated in ethanol-fed mice compared to pair-fed mice, but the elevation was eliminated by CR2-Crry treatment. Furthermore, ethanol-induced steatosis increased the susceptibility of livers to warm hepatic IRI (Fig. 2a, b). C3 was activated in the ethanol-fed mice, but C3 activation was inhibited by CR2-Crry treatment (Fig. 2c). In addition, TNF-α and IL-6 levels were increased in the ethanol-fed mice, and CR2-Crry treatment significantly reduced the levels of both cytokines (Fig. 2d, e). Thus, CR2-Crry treatment protects against liver IRI.

### Complement C3 regulates the expression of CYP2E1

**Fig. 3:**
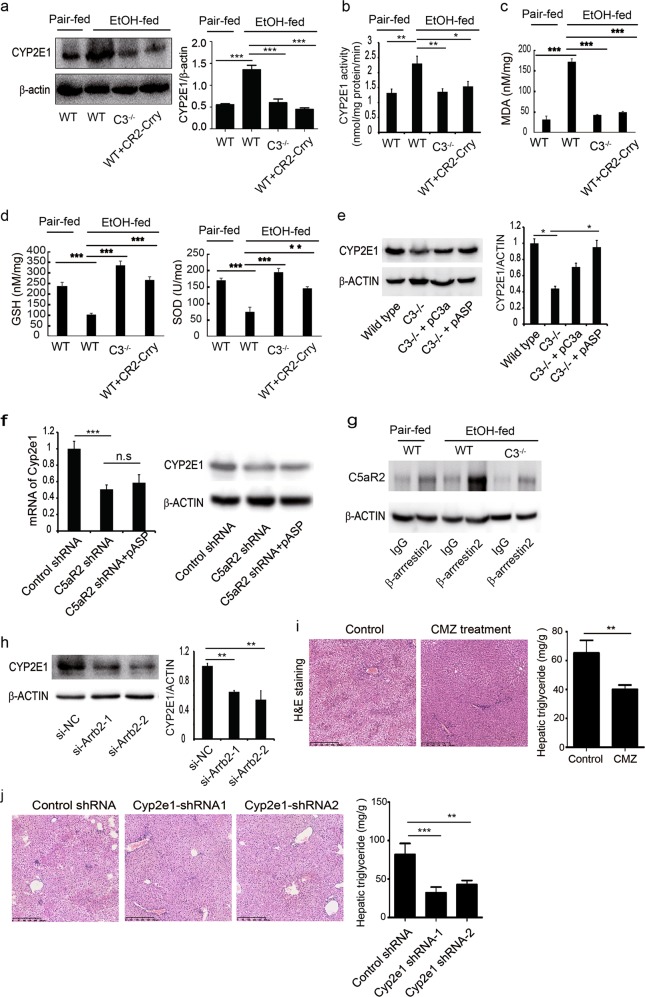
C3 contributes to the ethanol-induced steatosis by regulating CYP2E1 expression in AFLD mice. **a** The expression of CYP2E1 in CR2-Crry-treated or *C3*^−/−^ mice was detected by western blot. **b** CYP2E1 activity. **c, d** MDA, GSH and SOD levels in liver samples. **e** *C3*^−/−^ mice were administered with the peptide C3a (pC3a) or Asp (pAsp). Saline administration served as the control. The expression of CYP2E1 was detected by western blot. **f** AAV9-shRNAs were used to knock down *C5aR2*. The effect of C5aR2 knockdown on the expression of CYP2E1 was examined. **g** Co-immunoprecipitation of C5aR2 with β-arrestin2. **h** AML12 cells were transfected with *Arrb2* siRNA, and after 12 h treated with 100 mM ethanol. CYP2E1 expression was detected by western blot. NC, negative control for siRNA. **i, j** AAV9-shRNAs or CMZ was used to downregulate *Cyp2e1*. The effect of CYP2E1 downregulation on liver steatosis was determined by H&E stainging and liver triglyceride levels. The data are representative of three independent experiments. n.s., not significant. The results are expressed as means ± SD. *n* = 6, *P < 0.05, **P < 0.01, ***P < 0.001

Oxidative stress is a predominant factor that contributes to the pathogenesis of AFLD.^31,32^ We therefore investigated the effect of C3 deficiency or CR2-Crry treatment on the expression of CYP2E1, a member of the cytochrome P450 mixed function oxidase system. We found that the CYP2E1 expression and activity were increased in ethanol-fed WT mice, but this effect was abolished in *C3*^−/−^ or CR2-Crry-treated mice (Fig. 3a, b). Furthermore, glutathione (GSH) and superoxide dismutase (SOD) levels were diminished in ethanol-fed mice, but their levels were recovered in *C3*^−/−^ or CR2-Crry-treated mice. In contrast, malondialdehyde (MDA) level was elevated in ethanol-fed mice, but C3 deficiency or CR2-Crry treatment reduced the MDA level (Fig. 3c, d). The decreased expression of CYP2E1 in *C3*^−/−^ mice was restored by treatment with the peptide C3a or its degraded form, C3a-des-Arg (also known as Asp), again indicating a causative link with C3 activation (Fig. 3e). Although not without controversy, C5aR2 is the only identified receptor for Asp. Notably, the expression of CYP2E1 decreased upon C5aR2 knockdown, and Asp treatment failed to restore the expression of CYP2E1 (Fig. 3f). Previous studies have shown that C5aR2 binds to β-arrestin2, thereby mediating other signaling pathways involved in transcriptional regulation of CYP2E1.^45,46^ Co-immuoprecipitation showed that β-arrestin2 binds to C5aR2 in liver tissue of ethanol-fed mice, which was reduced following the decrease of C3 stimulation in ethanol-fed *C3*^−/−^ mice (Fig. 3g). Furthermore, β-arrestin2 knockdown by siRNA resulted in the downregulation of CYP2E1 expression (Fig. 3h). In addition, adeno-associated virus 9 (AAV9)-shRNAs or CMZ, an inhibitor of CYP2E1, was used to downregulate *Cyp2e1* (Supplementary information, Fig. S3a, b), and the effect on liver steatosis was evaluated. *Cyp2e1*-shRNAs or CMZ treatment lowered liver triglyceride levels and micro- and macro-vesicular fat accumulation in ethanol-fed mice (Fig. 3i, j).

### C3 but not C5 regulates Gly-tRF expression contributing to steatosis in AFLD mice

**Fig. 4:**
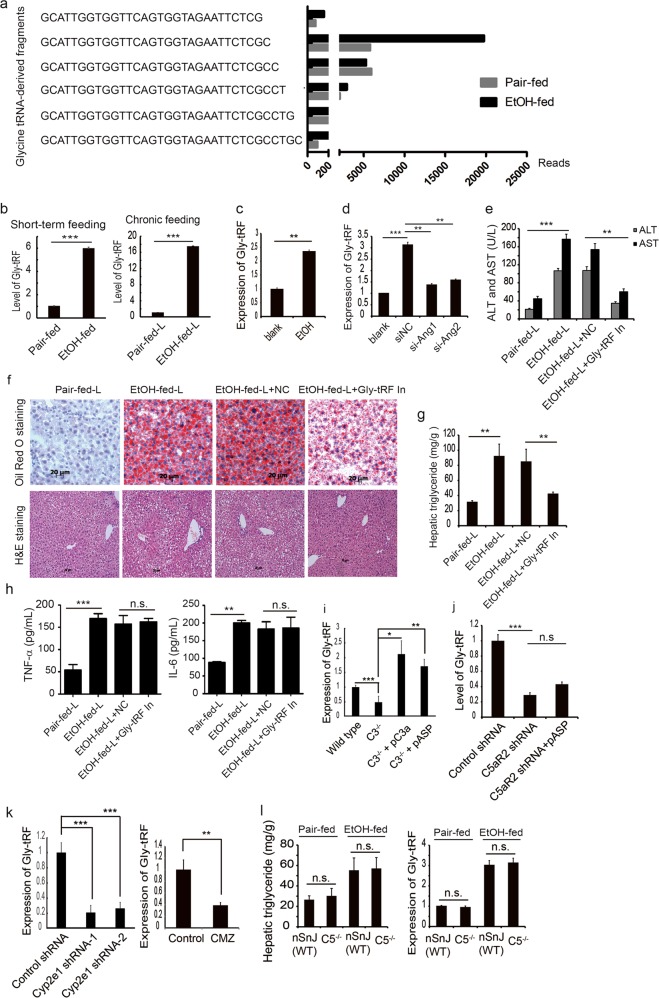
Gly-tRFs are overexpressed in ethanol-fed mice and promote liver steatosis. **a** Sequence alignment analysis of glycine tRNA-derived fragments. Gly-tRFs were upregulated in the liver of ethanol-fed mice. The data is representative of two independent experiments. **b** Hepatic expression of Gly-tRF in a short-term feeding or chronic feeding model as detected by qRT-PCR. Pair-fed-L or EtOH-fed-L represents chronic feeding. **c** AML12 cells were cultured with DMEM-F12 and treated with 100 mM ethanol. Gly-tRF expression was detected by qRT-PCR. U6 was used as an internal control for RNA loading. **d** AML12 cells were transfected with ANG siRNA, and after 12 h treated with 100 mM ethanol. Gly-tRF expression was detected by qRT-PCR. NC, negative control for siRNA. **e** Chronic ethanol-fed mice (*n* = 6) were injected i.p. with Gly-tRF antisense inhibitors. Serum ALT and AST levels were detected. **f** Frozen liver sections stained with Oil Red O and paraffin-fixed liver sections stained with H&E. **g** Liver triglyceride levels. **h** Serum TNF-α and IL-6 levels. **i** *C3*^−/−^ mice were administered with the peptide C3a or Asp. Saline administration served as the control. The expression of Gly-tRF was examined by qRT-PCR. **j** AAV9-shRNAs were used to knock down *C5aR2*. The effect of C5aR2 knockdown on the expression of Gly-tRF was examined. **k** The effect of CYP2E1 downregulation on Gly-tRF expression was analyzed. **l** C5 deficient and haplotype mice were subjected to short-term binge feeding. The effect of C5 deficiency on steatosis or the expression of Gly-tRF was examined. The data are representative of three independent experiments. n.s., not significant. The results are expressed as the means ± SD. *P < 0.05, **P < 0.01, ***P < 0.001

Oxidative stress plays an important role in the development of AFLD, and ROS levels in the liver increase following alcohol consumption. tRNA cleavage is reportedly conserved in response to oxidative stress,^29^ and we hypothesized that a tRNA-derived fragment is a contributor to the development of AFLD. We performed small RNA high-throughput sequencing of hepatic tissues from short-term ethanol-fed mice followed by bioinformatics analysis. Small RNAs, but not microRNAs, can be generated from novel RNA sources such as tRNAs and rRNAs. We found that Gly-tRFs were upregulated in the liver of the ethanol-fed mice compared to the pair-fed mice (Fig. 4a). Sequence alignment analysis suggests that highly expressed Gly-tRFs (29–34 nt) were cleaved from the 5′ end of the tRNA precursors at the anticodon loop (Fig. 4a). Gly-tRF levels were verified by quantitative reverse transcription PCR (qRT-PCR), and as shown in Fig. 4b, short-term ethanol feeding increased the hepatic expression of Gly-tRF. Notably, the hepatic expression of Gly-tRFs was significantly upregulated in chronic ethanol-fed mice compared to pair-fed mice. To investigate whether Gly-tRF expression is induced by ethanol, AML12 liver cells were cultured in the presence of ethanol. Gly-tRF expression was increased by treatment with ethanol (Fig. 4c). We also evaluated the effect of H_2_O_2_ on Gly-tRF expression in AML12 cells, and found that H_2_O_2_ induced a dose-dependent increase in Gly-tRF levels (Supplementary information, Fig. S3c). Several studies have indicated that ANG induced by stress is involved in the biogenesis of tRFs.^23,25^ We used siRNA to knock down *Ang*, and its attenuation was validated by qRT-PCR and western blot analyses (Supplementary information, Fig. S3d). ANG downregulation decreased Gly-tRF expression after treatment with ethanol or H_2_O_2_, as determined by qRT-PCR (Fig. 4d; Supplementary information, Fig. S3e).

To investigate the role of Gly-tRF in AFLD, we used anti-sense inhibitors to block Gly-tRF expression in vivo in the AFLD mouse model. Compared to the controls, serum ALT and AST levels were significantly reduced in the Gly-tRF inhibitor treated mice (Fig. 4e). Oil Red and H&E staining showed that treatment with Gly-tRF inhibitors significantly decreased ethanol-induced micro- and macro-vesicular hepatic steatosis (Fig. 4f, g). However, the proinflammatory cytokines TNF-α and IL-6 were not affected by Gly-tRF inhibition (Fig. 4h). As described above, complement C3 regulates the expression of CYP2E1 in AFLD mice. Thus, we analyzed the expression of Gly-tRF in ethanol-fed *C3*^−/−^ mice or in CR2-Crry-treated mice. Gly-tRF expression was significantly decreased in *C3*^−/−^ mice or CR2-Crry-treated mice compared to untreated WT mice (Supplementary information, Fig. S3f). Furthermore, the expression of Gly-tRF in *C3*^−/−^ mice was restored by treatment with peptide C3a or Asp, indicating a causative link (Fig. 4i). Knockdown of C5aR2 by AAV9-shRNA led to a decrease in the expression of Gly-tRF in AFLD mice, which was not restored by Asp (Fig. 4j). Furthermore, downregulation of CYP2E1 by shRNAs or CMZ decreased the hepatic expression of Gly-tRF in AFLD mice (Fig. 4k). Interestingly, C5 deficiency had little effect on the expression of Gly-tRF and liver steatosis in ethanol-fed mice (Fig. 4l), indicating that complement activation products downstream of C5 cleavage/activation are not involved in these pathogenic mechanisms.

### Gly-tRF is involved in regulation of lipid metabolism pathway in AFLD mice

**Fig. 5:**
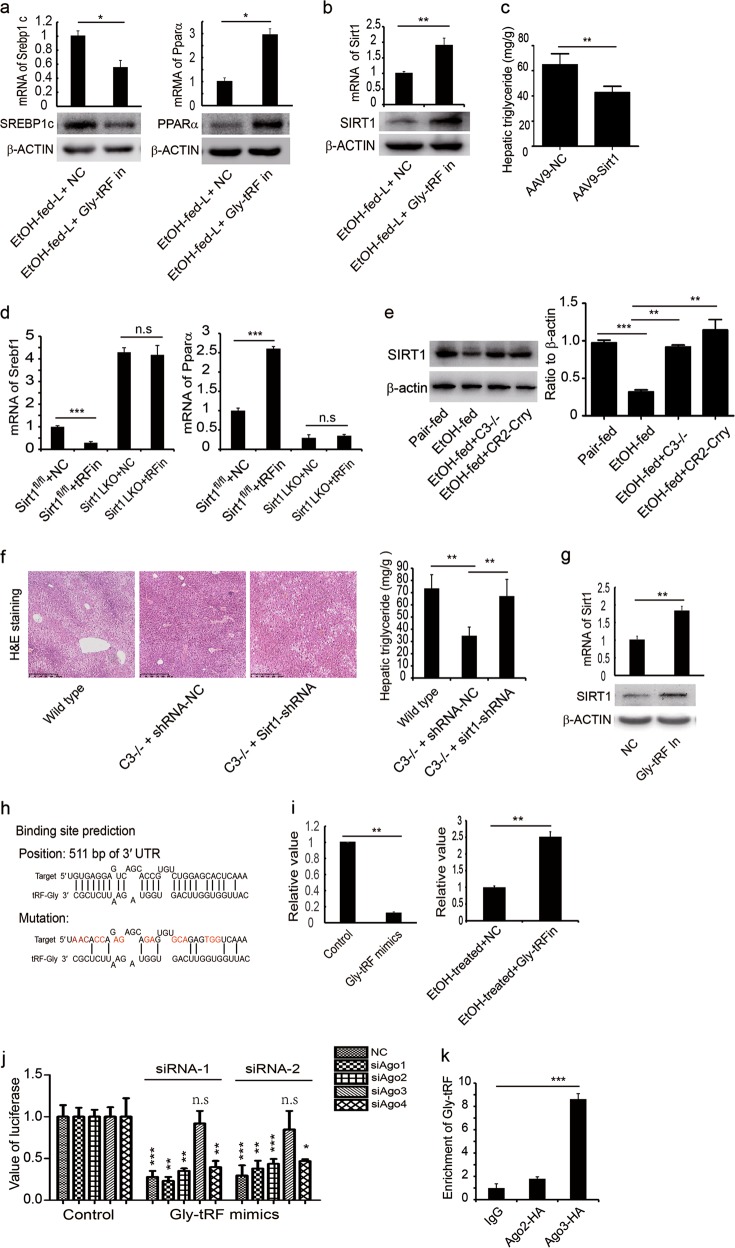
Gly-tRF is involved in regulation of lipid metabolism pathway. **a** Hepatic expression of *Srebp-1c* or *Pparα* was detected by qRT-PCR and western bot. **b** Hepatic mRNA and protein levels of SIRT1 were detected by qRT-PCR and western blot, respectively. **c** Hepatic triglyceride levels were analyzed upon overexpression of *Sirt1* by AAV9-*Sirt1*. **d** Ethanol-fed Sirt1^fl/fl^ and Sirt1 LKO mice were treated with the Gly-tRF ihnibitor or control. The expression of *Srebp1* and *Ppara* was examined by qRT-PCR. **e** The expression of SIRT1 was detected by western blot in pair-fed, and ethanol-fed WT, *C3*^−/−^ and CR2-Crry-treated mice. **f** Effect of *Sirt1* knockdown on the liver steatosis in the ethanol-fed *C3*^−/−^ mice. **g** AML12 cells were transfected with Gly-tRF antisense inhibitors or corresponding controls. The expression of Sirt1 was detected by qRT-PCR and western blot. Random sequence was used as control for inhibitors. **h** Predicted binding site in the *Sirt1* 3′ UTR. A mutation at binding site was generated by PCR mutagenesis. **i** AML12 cells were co-transfected with plasmid expressing *Sirt1* 3′ UTR and Gly-tRF mimics, or antisense inhibitors with treatment of 100 mM ethanol. After 48 h cells were collected for luciferase assays. **j** AML12 cells were co-transfected with plasmid expressing *Sirt1* 3′ UTR, Gly-tRF mimics, and siRNAs targeting *Ago1*, *Ago2*, *Ago3*, *Ago4*. After 48 h cells were collected for luciferase assays. **k** AML12 cells were transfected with plasmid expressing AGO3-HA or AGO2-HA, or vector control. The enrichment of gly-tRF was detected by RIP-qPCR. The data are representative of three independent experiments. Results are expressed as the means ± SD. *n* = 6, *P < 0.05, **P < 0.01, ***P < 0.001. n.s., not significant. EtOH-fed-L, chronic ethanol-fed mice; NC, negative control; Gly-tRF In, Gly-tRF antisense inhibitors

We next investigated the molecular mechanism associated with Gly-tRFs in the AFLD mouse model. Hepatic tissues from mice treated with Gly-tRF inhibitors were subjected to transcriptome sequencing, and Gly-tRF was shown to be associated with lipid metabolism in AFLD mice (Supplementary information, Fig. S4a). Genes related to fatty acid synthesis were downregulated, whereas β-oxidation-associated genes were upregulated by treatment with Gly-tRF antisense inhibitors (Supplementary information, Table S2). The results of qRT-PCR and western blot showed that hepatic expression of fatty acid synthesis related genes such as *Srebp-1c*, *Fasn*, *Lipin1* and *Acc* was downregulated by Gly-tRF inhibitors (Fig. 5a; Supplementary information, Fig. S4b). In contrast, the expression of the β-oxidation-related genes *Pparα* and *Cpt1a* was upregulated (Fig. 5a; Supplementary information, Fig. S4c). In addition, the expression of *Cyp2e1* and *Ang* was decreased by Gly-tRF inhibitor treatment (Supplementary information, Fig. S4d). Interestingly, transcriptome profiling indicated that Gly-tRF inhibitors upregulate the in vivo hepatic expression of *Sirt1* (Supplementary information, Table S2). Previous studies have indicated that ethanol-induced attenuation of hepatic SIRT1 plays an important role in the pathogenesis of AFLD, and that stimulation of SIRT1 expression protected against the development of AFLD.^34,36,47,48^ We validated the hepatic expression of *Sirt1* by qRT-PCR and western blot analyses, which showed that treatment with Gly-tRF inhibitors increased *Sirt1* expression in hepatic tissues (Fig. 5b). Notably, the overexpression of *Sirt1 via* AAV9-*Sirt1* alleviated liver steatosis in the AFLD mice (Fig. 5c; Supplementary information, Fig. S5a, b). Although the transcriptional level of C3 was not affected by *Sirt1* overexpression, the activation level of C3 was decreased (Supplementary information, Fig. S5c). The expression of CYP2E1, ANG and Gly-tRF was downregulated by the overexpression of *Sirt1* (Supplementary information, Fig. S5d, e). *Srebp1* expression was downregulated by the overexpression of *Sirt1*, whereas the expression of *Cpt1a* was upregulated (Supplementary information, Fig. S5f), indicating that SIRT1 is a critical regulator of lipid metabolism pathways. We further used SIRT1 liver-specific knockout (LKO) mice to study the effect of Gly-tRF inhibitors. Treatment with Gly-tRF inhibitors downregulated the expression of *Srebp1* and upregulated the expression of *Ppara* in ethanol-fed Sirt1^fl/fl^ mice, but failed to further downregulate the expression of *Srebp1* or to upregulate *Ppara* expression in ethanol-fed SIRT1 LKO mice (Fig. 5d). These results indicate that Gly-tRF regulates lipid metabolism by targeting *Sirt1* in AFLD mice. Because C3 regulates the expression of Gly-tRF, we investigated whether C3 activation affects the expression of SIRT1 via mediating the expression of Gly-tRF. We found that SIRT1 expression was restored in C3-deficient or CR2-Crry-treated mice compared to untreated ethanol-fed mice (Fig. 5e). In addition, the protective effects of C3 deficiency was reversed by the knockdown of *Sirt1* in the AFLD mice (Fig. 5f). These results indicate that Gly-tRF regulates lipid metabolism by targeting *Sirt1* in AFLD mice.

To further explore the molecular mechanism of Gly-tRF regulation of *Sirt1* expression, AML12 cells were transfected with Gly-tRF mimics, inhibitors or corresponding controls, and after 48 h the cells were analyzed by qRT-PCR and western blotting. *Sirt1* expression was decreased in the presence of Gly-tRF mimics, but was upregulated by Gly-tRF inhibitors in AML12 cells (Fig. 5g; Supplementary information, Fig. S6a). We then predicted the binding sites of Gly-tRF in the *Sirt1* 3′ UTR using online software (Fig. 5h). The *Sirt1* 3′ UTR sequence was cloned into the pMir-reporter vector, and the construct was co-transfected with Gly-tRF mimics, inhibitors or corresponding controls into AML12 cells, respectively. After 48 h, cells were collected for luciferase assays. The results showed that luciferase expression decreased after treatment with Gly-tRF mimics, but increased with Gly-tRF inhibitors (Fig. 5i). Next, a mutation was introduced into the predicted binding sites (Fig. 5h), and a mutant-containing plasmid was co-transfected with Gly-tRF mimics, inhibitors or corresponding controls, respectively. The binding site mutation inhibited downregulation of luciferase expression by Gly-tRF or upregulation by Gly-tRF inhibitors (Supplementary information, Fig. S6b). In addition, when *Sirt1* was downregulated by AAV9-shRNAs, Gly-tRF inhibitor did not further ameliorate liver steatosis in mice (Supplementary information, Fig. S6c, d). Studies have shown that AGO family proteins associate with tRFs that are involved in regulation of gene expression.^18^^,^^49–51^ Therefore, we used siRNAs to knock down *Ago1*, *Ago2*, *Ago3*, or *Ago4* (Supplementary information, Fig. S6e), and revealed that luciferase expression was not affected by the silencing of *Ago1*, *Ago2* and *Ago4*. However, upon *Ago3* knockdown, Gly-tRF no longer inhibited the expression of luciferase (Fig. 5j). RIP-qPCR analysis indicated that AGO3, but not AGO2, significantly enriched Gly-tRFs (Fig. 5k). These results suggest that AGO3 mediates Gly-tRF regulation of gene expression.

### The expression of C3d, CYP2E1, Gly-tRF and *Sirt1* in AFLD patients

**Fig. 6:**
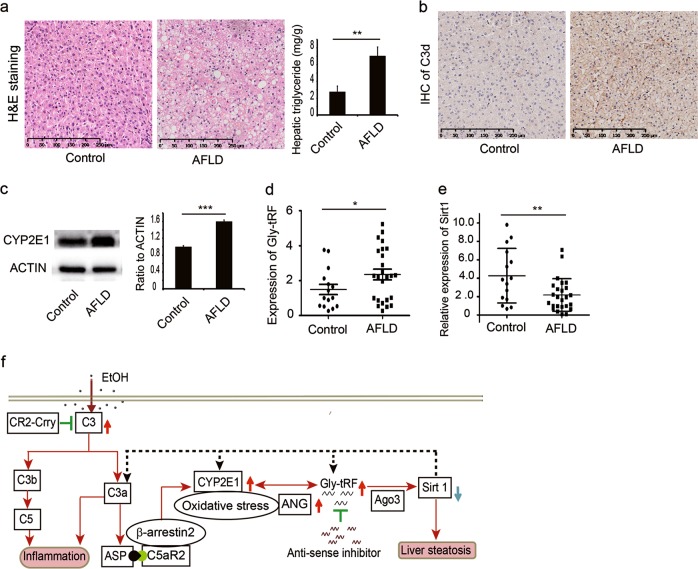
The expression of C3d, CYP2E1, Gly-tRF and *Sirt1* in AFLD patients. **a** H&E staining and hepatic triglyceride levels. **b** Immunohistochemistry (IHC) of C3d. **c** The expression of CYP2E1. **d, e** The expression of Gly-tRF and *Sirt1* in liver tissues of AFLD patients (*n* = 30) and healthy controls (*n* = 15) was detected by qRT-PCR. The data are representative of three independent experiments. The results are expressed as the means ± SD. *P < 0.05, **P < 0.01, ***P *<* 0.001. **f** Schematic graph showing that C3 mediates the expression of Gly-tRF contributing to the development of liver steatosis. Schematic graph illustrating a working model for the complement-mediated regulation of Gly-tRF expression in AFLD, and the potential therapeutic targets for the management of the disease. CR2-Crry is a site-targeted complement inhibitor of C3 activation. Asp is the degraded product of C3a. Anti-sense inhibitor is Gly-tRF inhibitor

Although the therapeutic effects of CR2-Crry or Gly-tRF inhibitors in ethanol-fed mice indicate an underlying mechanism for the development of AFLD, whether these findings point to a potential strategy for treating AFLD requirs validation in human specimens. We therefore investigated the expression of C3d, CYP2E1, Gly-tRF and *Sirt1* in the liver tissues of AFLD patients and healthy controls. Fat accumulation and liver triglyceride level increased in AFLD patients compared to healthy contols (Fig. 6a), and C3d accumulated in AFLD patients relative to controls (Fig. 6b). Notably, the levels of CYP2E1 and Gly-tRF were upregulated in AFLD patients compared to healthy controls (Fig. 6c, d). We further examined the expression of *Sirt1* and showed that *Sirt1* expression significantly decreased in liver tissues of AFLD patients compared to healthy controls (Fig. 6e). These results are consistent with our animal studies, indicating a potential clinical approach.

Taken together, the data elucidate the molecular mechanisms whereby C3 regulates liver steatosis and inflammation. Both C3a and Asp promote the expression of Gly-tRFs via mediating the expression of CYP2E1, which contributes to the development of liver steatosis by regulating *Sirt1* expression (Fig. 6f).

## Discussion

In this study, we investigated the related mechanisms of how complement contributes to AFLD and explored the therapeutic potential of a site-targeted complement inhibitor. We found that C3 activation is involved in regulation of both hepatosteatosis and inflammatory responses and contributes to liver IRI in AFLD mice. CR2-Crry treatment significantly reduced inflammation and hepatic steatosis in AFLD mice, in addition to alleviating apoptosis and liver IRI. Our data indicate that C3 activation products regulate the expression of CYP2E1 *via* the signaling of β-arrestin2. We show that expression of Gly-tRFs is upregulated in both ethanol-fed mice and AFLD patients. Inhibition of Gly-tRFs in vivo resulted in a reduction in hepatic steatosis, but not in inflammatory response in ethanol-fed mice. The expression of Gly-tRF was dowregulated in *C3*^−/−^ mice or CR2-Crry-treated WT mice, which was restored by the C3 activation products C3a or Asp, indicating a novel mechanism by which C3 contributes to the development of hepatosteatosis. Futhermore, Gly-tRFs interact with AGO3 to downregulated *Sirt1* expression *via* a complementary sequence in the 3′ UTR. As a result, fatty acid synthesis and lipid oxidation pathways are altered, leading to liver steatosis and injury.

Currently, there are few effective drugs approved for the treatment of AFLD. Previous studies have shown that C3 and C5 play different roles in the progression of AFLD.^4^ C3 deficiency protects against ethanol-induced liver injury and steatosis. In contrast, *C5*^−/−^ mice still exhibit hepatosteatosis, but have lower inflammatory cytokines levels in the AFLD mouse model.^4^ In this study, we assessed the therapeutic effect of the site-targeted complement inhibitor, CR2-Crry, on AFLD in mouse models. CR2-Crry inhibits C3 convertase and thus suppresses C3 cleavage and activation, leading to a reduction in both inflammatory cytokine levels and hepatic steatosis after ethanol feeding. These results suggest that C3 inhibitors, in particular a humanized form of CR2-Crry, may provide an effective treatment for AFLD patients with both inflammatory response syndrome and hepatic steatosis. In addition, patients with advanced forms of AFLD usually require surgical intervention to improve survival. IRI is an inevitable consequence of surgical procedures, and complement activation plays a key role in hepatic IRI.^8,9^ Our results indicate that targeted C3 inhibition may provide a therapeutic strategy to protect against hepatic IRI for severe AFLD patients.

Oxidative stress is a typical feature of ethanol-induced AFLD, with ethanol degradation catalyzed by cytochrome P450 enzymes, including CYP2E1, as part of a major pathway. A previous study reported that hepatic steatosis is elevated in CYP2E1 knock-in mice after ethanol feeding.^52^ Pritchard et al.^4^ reported that ethanol feeding increases the expression of CYP2E1 in both WT and *C3*^−/−^ mice compared to pair-fed mice. In the present study, however, CYP2E1 expression was reduced in ethanol-fed *C3*^−/−^ or CR2-Crry-treated WT mice compared to ethanol-fed WT mice. Furthermore, the expression of CYP2E1 in *C3*^−/−^ mice was restored by the treatment with C3a or Asp. Currently, C5aR2 is the only identified receptor for Asp,^53,54^ and C5aR2 plays a critical role in triglyceride synthesis.^55^ Knockdown of C5aR2 by AAV9-shRNAs reduced the expression of CYP2E1, which was not restored by Asp, indicating that C5aR2 is a key regulator of the development of AFLD. Previous studies have shown that C5aR2 binds to β-arrestin2,^45^ which acts as scaffolds for the β-catenin or JNK activation,^46^ and the β-catenin or JNK pathway is involved in transcriptional regulation of CYP2E1.^56,57^ Our studies indicate that Asp/C5aR2 regulates the expression of CYP2E1 through the β-arrestin2 signaling pathway.

The contribution of C3 to hepatosteatosis has been previously investigated. However, the mechanisms by which complement is involved in ethanol-induced steatosis are poorly understood. Small RNA high-throughput sequencing of hepatic tissues indicated that Gly-tRFs are upregulated in ethanol-fed mice compared to pair-fed mice. Previous studies have indicated that tRFs play critical roles in cellular processes and carcinogenesis.^14,16^ However, a role for Gly-tRFs in AFLD has not been previously described. In this study, Gly-tRF antisense inhibitor treatment significantly reduced hepatic steatosis in AFLD mice, but did not affect the inflammatory response. These results suggest that Gly-tRF was associated with the development of liver steatosis. In addition, C3 deficiency or CR2-Crry treatment decreased the expression of Gly-tRF. Furthermore, the expression of Gly-tRF in *C3*^−/−^ mice was restored by administration of mice with the peptides C3a or Asp. Notably, C5 deficiency had little effect on the development of liver steatosis and the expression of Gly-tRF in AFLD mice. Several lines of evidence have shown that C3 activation contributes to both liver steatosis and inflammatory response, however, C5-deficient AFLD mice still exhibit hepatosteatosis but reduced inflammatory response.^4^ These studies further support that Gly-tRF is involved in regulation of liver steatosis but not inflammatory response. Following the generation of C3a, a serum carboxypeptidase rapidly cleaves its C-terminal Arg residue to form a more stable Asp. We found that C3a and Asp function similarly to regulate the expression of Gly-tRF. Notably, downregulation of CYP2E1 by shRNAs or CMZ decreased the expression of Gly-tRF in ethanol-fed mice. Taken together, C3a/Asp is a driving factor to regulate the expression of Gly-tRF through the CYP2E1 pathway. Chronic alcohol intake leads to AFLD, which encompasses a broad spectrum of diseases, including simple steatosis, steatohepatitis, fibrosis, and cirrhosis. More than 90% of heavy drinkers develop AFLD. Interventional therapy is conducted in this critical stage of liver steatosis, which may protect against the development of severe forms of AFLD. Together, these results indicate that inhibitors of Gly-tRF may be used for a potential precision therapy for patients with hepatosteatosis.

Our transcriptome sequencing data revealed that *Sirt1* expression increases upon Gly-tRF antisense inhibitor treatment. Our results indicate that Gly-tRFs regulate *Sirt1* expression in hepatic tissue of mice, which was further confirmed *in vitro* using AML12 cells. Studies have shown that SIRT1 can regulate diverse lipid metabolism pathways in the liver,^36^ including lipogenesis and fatty acid β-oxidation. Furthermore, hepatic SIRT1 signaling pathways play a predominant role in AFLD development. Hepatic deletion of *Sirt1* facilitates the development of hepatic steatosis in response to ethanol feeding in mice, mainly by altering the function of LIPIN1, a transcriptional regulator of lipid metabolism.^38^ In addition, SIRT1 is a key regulator of *Pparα* signaling, which is the major regulator stimulating hepatic mitochondrial β-oxidation of fatty acids. Our transcriptome profiling data showed that genes related to fatty acid synthesis such as *Srebp-1c*, *Lipin1*, and *Fasn* were downregulated, whereas the *Pparα* and *Cpt1a* genes that are associated with fatty acid β-oxidation were upregulated by Gly-tRF inhibitors. Furthermore, the results of binding site mutations demonstrate that Gly-tRFs downregulate *Sirt1* expression *via* 3′ UTR sequence complementarity. These results indicate that regulation of the SIRT1 pathways by Gly-tRFs represents a molecular mechanism for the development of ethanol-induced hepatic steatosis. These findings reveal a novel mechanism for regulating the expression of *Sirt1*, and indicate the Gly-tRF inhibition is a potential strategy for the restoration of ethanol-mediated impairment of SIRT1 in AFLD.

Several lines of evidence indicate that the complement system is activated in AFLD patients. Wlazlo et al.^58^ reported that activated C3 is associated with AFLD, and the immunoreactive intensity of C1q, C3, and C5 in patients with alcoholic hepatitis is significantly higher than that seen in normal controls.^59^ In addition, previous studies have shown that hepatic CYP2E1 can be induced by ethanol consumption and that CYP2E1 levels are higher in patients with AFLD.^60–62^ Interestingly, serum SIRT1 was downregulated in AFLD patients compared with healthy controls.^63^ Moreover, the mRNA level of *Sirt1* was also lower in an AFLD group compared to a control group.^63^ Our study shows that C3d, CYP2E1 and Gly-tRF are upregulated, but that *Sirt1* is downregulated, in AFLD patients compared to healty controls. These results are consistent with previous preclinical studies, indicating a potential therapeutic strategy for AFLD. Previous studies indicated that tRFs with different lengths processed from the same tRNA have different functions.^64,65^ In this study, we focused on the tRF with a length of 29–34 nt, which is fundamentally distinct from tRFs of ~22 nt in length.^64^ In addition, recent studies showed that RNA modifications are associated with the function of tRFs in sperm or stem cells.^19,20^ RNA modification of tRF in somatic cells is incompletely understood, and is an area for future study.

In summary, our results demonstrate that complement C3 inhibition mitigates the severity of early stage AFLD. We found that the C3 activation products C3a and Asp play key roles in the development of hepatic steatosis by regulating the expression of Gly-tRF *via* CYP2E1. Our study reveals a novel mechanism by which Gly-tRFs promote lipogenesis and inhibit fatty acid β-oxidation through regulating the SIRT1 signaling pathway. These findings establish a working model encompassing the complement-mediated regulation of Gly-tRF expression in AFLD, and reveal promising therapeutic targets for the management of the disease. Notably, humanized versions of complement inhibitors may potentially be employed in the treatment of AFLD, and inhibitors of Gly-tRF represent a potential precision therapy strategy for the liver steatosis of AFLD.

## Materials and Methods

### Mice and human specimens

*C3*^−/−^ mice on C57BL/6 background (Stock# 003641), C57BL/6 WT, *C5*^−/−^ (B10.D2-*Hc*^*0*^*H2*^*d*^*H2-T18*^*c*^/0SnJ) and their haplotype (B10.D2-*Hc*^*1*^*H2*^*d*^*H2-T18*^*c*^/nSnJ), SIRT1^flox/flox^ (Stock# 029603) were purchased from the Jackson Laboratory (Bar Harbor, ME, USA). Alb-cre mice were kindly provided by the Center of Shanghai Model Organisms. All liver tissue samples were collected from clinically defined AFLD patients and healthy controls as part of an approved institutional review board (IRB). Healthy liver tissue was obained from liver donation or benign liver diseases such as liver hemangioma. All animal experiments were approved by the Animal Care and Use Committee of Guangxi Medical University, Guangxi, China.

### Ethanol feeding protocols

Eight- to ten-week-old female C57BL/6 WT, *C3*^−/−^ mice and littermate WT mice were separately subjected to gavage, short-term binge feeding or chronic feeding. Eight- to ten-week-old female *C5*^−/−^ and their haplotype mice were subjected to short-term binge feeding. The ethanol feeding protocols were modified from a previously described approach: 1) Ethanol gavage^4^: mice were gavaged with a single dose of ethanol (6 g/kg body weight, 31.5% ethanol), and PBS or CR2-Crry (dose: 0.25 mg) was injected intraperitoneally (i.p.) 30 min before gavage. 2) Short-term binge feeding^66^: mice were fed a control Lieber-DeCarli diet for five days to acclimatize them to a liquid diet, then allowed free access to the Lieber-DeCarli diet containing 5% (v/v) ethanol for 10 days; control groups were pair-fed with an isocaloric control diet. The mice were gavaged with a single dose of ethanol (6 g/kg body weight) or isocaloric dextrin-maltose on the 16^th^ day and euthanized 9 h later. PBS or CR2-Crry (dose: 0.25 mg) was injected i.p. every other day for the last six days, and an additional injection was given 30 min before gavage. This protocol for chronic-plus-single-binge ethanol feeding synergistically induced both steatosis and inflammation, which mimics acute-on-chronic alcoholic liver injury in patients.^66^ We used this model to study the effect of complement inhibitor CR2-Crry on both steatosis and inflammation of ALD. 3) Chronic feeding^4,67^: the mice were randomly assigned to either pair-fed or ethanol-fed groups. The mice were allowed free access to the control liquid diet for three days, then the ethanol-fed group was allowed free access to the increasing concentrations of ethanol in a complete liquid diet. Control mice were given pair-fed diets, and the ethanol-fed mice were given ethanol at concentrations as follows: 1% ethanol for two days followed by 2% for two days, 4% for seven days, and 5% for three weeks. Mean body weights were similar in all groups at the end of the experiments. The chronic feeding model can mimic the pathogenesis of alcoholic fatty liver, but only induces little inflammation response. We used this model to investigate the role of Gly-tRF in liver steatosis via antisense inhibitors.

### Small RNA high-throughput sequencing

Total RNA was extracted from hepatic tissues of short-term ethanol-fed and pair-fed mice with TRIzol reagent (Invitrogen, Carlsbad, CA, USA). The quality of the RNA samples was evaluated with a NanoDrop 2000c spectrophotometer (Thermo Fisher Scientific, Waltham, MA, USA) and Agilent’s bioanalyzer, and RNA samples were size-fractionated with 15% polyacrylamide gel electrophoresis (PAGE) to collect the 18–40-nt fractions. Sequencing libraries were generated by RT-PCR amplification. The PCR products were purified and sequenced on a HiSeq 2500 sequencing system (BGI Tech Solutions, Shenzhen, China). High-throuhghput sequencing data can be found at the GEO database in NCBI (accession number: GSE126047).

### In vivo studies of Gly-tRF inhibitor

Mice were subjected to chronic feeding (1% ethanol for two days followed by 2% for two days, 4% for one week, and 5% for three weeks) according to the previously described approach.^4,67^ Synthetic Gly-tRF antisense inhibitors “CGUAAGCGAGAAUUCUACCACUGAACCACCAAUGCACAAU” or normal control (modified with 2′-O-Me and cholesterol; RiboBio, Guangzhou, China) (20 nmoles/per mouse) were injected i.p. into ethanol-fed mice twice weekly for the last three weeks. After three weeks, mice were euthanized and the effect of the Gly-tRF inhibitors on the liver was assessed. Hepatic tissues from mice treated with Gly-tRF inhibitors or normal controls (scrambled sequence) were subjected to transcriptome sequencing.

### Adeno-associated virus construction and infection

AAV9 was used to construct the shRNAs targeting the *C5aR2*, *Cyp2e1*, and *Sirt1* genes. AAV9-*Sirt1* was developed for overexpression of *Sirt1* (GeneChem, Shanghai, China). Recombinant virus (1.5 × 10^11^ v.g) was diluted in 0.2 mL of a saline solution and injected into the tail vein of mice. One week post injection, the mice were subjected to a short-term binge feeding.

### Peptides and chemical reagent

Mice were subjected to short-term binge feeding. During feeding with 5% ethanol, 0.1 mL of 150 μg/mL C3a peptide (pC3a) or Asp peptide (pAsp, also known as C3a-des Arg) was daily admininstered i.p. to the experimental group.^8^ Saline administration served as the control. On day 10, the mice were sacrificed, and liver tissues and serum were harvested. The C3a and Asp peptides were produced by Nanjing Peptide Industry (Nanjing, China). For the chlormethiazole (CMZ) experiment, CMZ (Sigma, 50 mg/kg body weight) was injected i.p. every other day at the time of 5% ethanol diet feeding.^68^

### Cell culture and transfection

AML12 cells were kindly provided by Stem Cell Bank, Chinese Academy of Sciences, Shanghai, China. The AML12 cells were cultured in Dulbecco’s modified Eagle medium (DMEM-F12) supplemented with 10% fetal bovine serum, 0.1 μM dexamethasone, and insulin-transferrin-selenium. The cells were grown at 37 °C in an atmosphere of 5% CO_2_. Gly-tRF mimics “GCATTGGTGGTTCAGTGGTAGAATTCTCGC” or normal control (modified with 2′-O-Me and cholesterol) was synthesized by RiboBio (Guangzhou, China). The AML12 cells were transfected with Gly-tRF mimics, Gly-tRF inhibitors, or small interfering RNA (siRNA) (RiboBio, Guangzhou, China) using Lipo3000 (Thermo Fisher Scientific, Waltham, MA, USA) according to the manufacturer’s protocol.

### Histopathologic and biochemical analyses

Fresh liver tissues were frozen, and sections (8-μm thick) were prepared and stained with Oil Red O. Other liver tissues were fixed with 10% neutral formalin and embedded in paraffin, and tissue sections (5-μm thick) were cut and stained with H&E. Histopathological alterations of the liver tissue were observed using a microscope. Plasma ALT and AST levels were measured with an autoanalyzer (ANTECH Diagnostics, Los Angeles, CA, USA).

### Immunohistochemistry

The resected liver tissues were fixed overnight with 10% formalin, and paraffin-embedded sections (4-μm thick) were prepared. Sections were then processed for immunohistochemical staining. Antigen retrieval was performed by pressure cooking for 3 min in citrate buffer (pH 6), followed by peroxidase and serum blocking steps. The sections were incubated with goat anti-C3d antibody (R&D Systems, 1:100 dilution) for 2 h at room temperature, followed by antibody detection with an anti-goat ImmPRESS kit (Vector Laboratories). Apoptosis was quantified by terminal deoxynucleotidyl transferase dUTP nick end labeling (TUNEL) using a TUNEL kit (Roche, Mannheim, Germany) according to the manufacturer’s instructions. The cell nuclei were counterstained with DAPI (1 mg/mL). The apoptotic cells were labeled green with the TUNEL staining kit. The images were collected using a fluorescent microscope (IX-71; Olympus, Tokyo, Japan).

### RNA extraction and qRT-PCR

Total RNA was extracted from hepatic tissues or AML12 cells according to the TRIzol extraction protocol (Invitrogen). Gly-tRFs were assayed according to a previously described method.^13^ RNA transcript levels were measured by qRT-PCR, using a SYBR Green PCR master mix (Bio-Rad). All experiments were performed in triplicate. The primers used in this study are shown in Supplementary information, Table S1.

### Measurement of SOD, GSH, MDA and triglyceride levels and CYP2E1 activity in liver homogenates

The levels of SOD, GSH, MDA and triglyceride in liver tissue homogenates from each group were measured using the corresponding kits (Catalog# A001-3, A006-2, A003-1, and A110-1, respectively) from Nanjing Jiancheng Bioengineering Institute (Nanjing, China) according to the manufacturer’s protocols. CYP2E1 activity was assayed by the rate of oxidation of *p*-nitrophenol to *p*-nitrocatechol according to a previously described protocol.^68^

### Western blot analysis

Proteins from hepatic samples or cells were analyzed by standard western blotting. Primary antibodies anti-SREBP1c (ab28481), anti-ANG (ab10600) and anti-CYP2E1 (ab28146) were from Abcam, Cambridge, UK; anti-PPARα (15540-1-ΑP) and anti-SIRT1 (13161-1-AP) were from Proteintech; anti-caspase-3 (#9662), anti-caspase-8 (#8592), anti-caspase-9 (#9504), anti-Bax (#2772), anti-Bcl2 (#3498), anti-STAT3 (#9139), anti-P-STAT3 (#9145), anti-Akt (#4685) and anti-P-Akt (#4060) were from Cell Signaling Technology. Anti-β-actin (66009-1-Ig; Proteintech) was used to normalize the signals.

### Serum cytokine and C3a measurements

Serum TNFα, IL-6 and C3a levels were measured using commercially available ELISA kits (R&D Systems) or (CUSABIO, Wuhan, China), following the manufacturer’s instructions.

### Plasmid construction and luciferase reporter assay

The 3′ UTR fragment of *Sirt1* was amplified using specific primers and cloned into the multiple cloning site of pMir-reporter vector (Promega, Madison, WI, USA). Mutations in the 3′ UTR fragment were generated *via* PCR-based mutagenesis. The primers used are shown in Supplementary information, Table S1. The AML12 cells were co-transfected with the plasmids and Gly-tRF mimics or inhibitors. After 48 h, the cells were collected and luciferase assays were performed according to the manufacturer’s protocol (Promega).

### RIP-qPCR and immunoprecipitation

AML12 cells were transfected with plasmids expressing AGO3-HA or AGO2-HA, or pcDNA3.1. Two days later, the AML12 cells were washed with cold PBS, placed on ice and irradiated with 254 nm UV light for 5 min.^69^ Next, the cells were collected and lysed in RIP buffer, following the manufacturer’s protocol (Magna RIP kit, Millipore). The cell lysates were then separated by centrifugation at 14,000 rpm for 15 min at 4 °C. Anti-HA antibody (M180-3, MBL) was mixed with magnetic beads coated with protein A/G and incubated with constant rotation for 30 min at room temperature. Subsequently, the anti-HA antibody and protein A/G-coated magnetic beads were mixed with the lysates and incubated at 4 °C for 4 h. The beads were then precipitated using a magnetic separator, followed by six washes with cool RIP buffer. The immunoprecipitated RNA samples were then extracted and reverse transcribed using an RT reagent kit (Invitrogen) with specifc primers, followed by qPCR. The results were normalized to input RNA levels and plotted as fold enrichment relative to that of the IgG control.

The liver tissues of pair-fed or ethanol-fed mice were disrupted using a lysis buffer containing a protease inhibitor mixture (Sigma) for 30 min on ice. The cell lysates were separated by centrifugation at 14,000 rpm for 15 min at 4 °C. Protein G/A-coated microbeads were incubated with the antibody β-arrestin2 (ab54790, Abcam) or IgG for 30 min at room temperature and then washed three times with cold lysis buffer. The microbeads were mixed with the cell lysates following incubation overnight at 4 °C. The immunoprecipitation samples were anlyzed by western blotting. Anti-C5aR2 (A10588, Abclonal) was used to detect endogenous C5aR2.

### Data analysis

Data were expressed as the means ± standard deviation (SD). Significant differences between groups were determined by ANOVA, with a Bonferroni correction for continuous variables and multiple groups. Two-tailed student’s *t-*test was used for the comparison of a normally distributed continuous variable between two groups. *P* values < 0.05 were considered statistically significant.

## Supplementary information

Supplementary information, Figure S1

Supplementary information, Figure S2

Supplementary information, Figure S3

Supplementary information, Figure S4

Supplementary information, Figure S5

Supplementary information, Figure S6

Supplementary information, Table S1

Supplementary information, Table S2
