# Supplementary material for: Complement C3 activation regulates the production of tRNA-derived fragments Gly-tRFs and promotes alcohol-induced liver injury and steatosis
Source: Cell Res. 2019 May 10;29(7):548–61. doi: 10.1038/s41422-019-0175-2 (PMC6796853; doi:10.1038/s41422-019-0175-2)
Supplement: Supplementary file 1 — Supplementary information, Figure S1 [file 41422_2019_175_MOESM1_ESM.pdf]

Supplementary information, Fig. S1

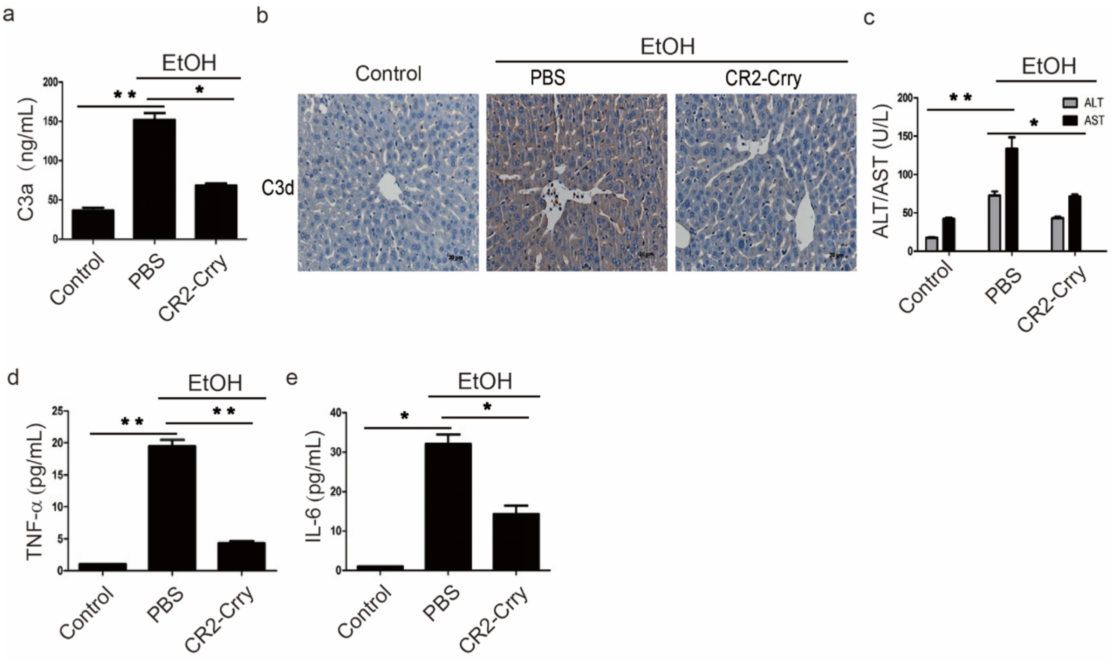

Fig. S1 Therapeutic effect of CR2-Crry on an acute AFLD model. Mice were subjected to acute ethanol gavage. Ethanol-fed mice were injected i.p. with PBS or CR2-Crry (0.25 mg/mouse). **a** Serum levels of C3a. **b** C3d deposition, 40×. **c** Serum levels of ALT and AST. **d** Serum levels of TNF-α. **e** Serum levels of IL-6. The data is representative of three independent experiments. The results are expressed as mean ± SD. $n = 6$, $^{*}P < 0.05$, $^{**}P < 0.01$.
