# Supplementary material for: Complement C3 activation regulates the production of tRNA-derived fragments Gly-tRFs and promotes alcohol-induced liver injury and steatosis
Source: Cell Res. 2019 May 10;29(7):548–61. doi: 10.1038/s41422-019-0175-2 (PMC6796853; doi:10.1038/s41422-019-0175-2)
Supplement: Supplementary file 2 — Supplementary information, Figure S2 [file 41422_2019_175_MOESM2_ESM.pdf]

Supplementary information, Fig. S2

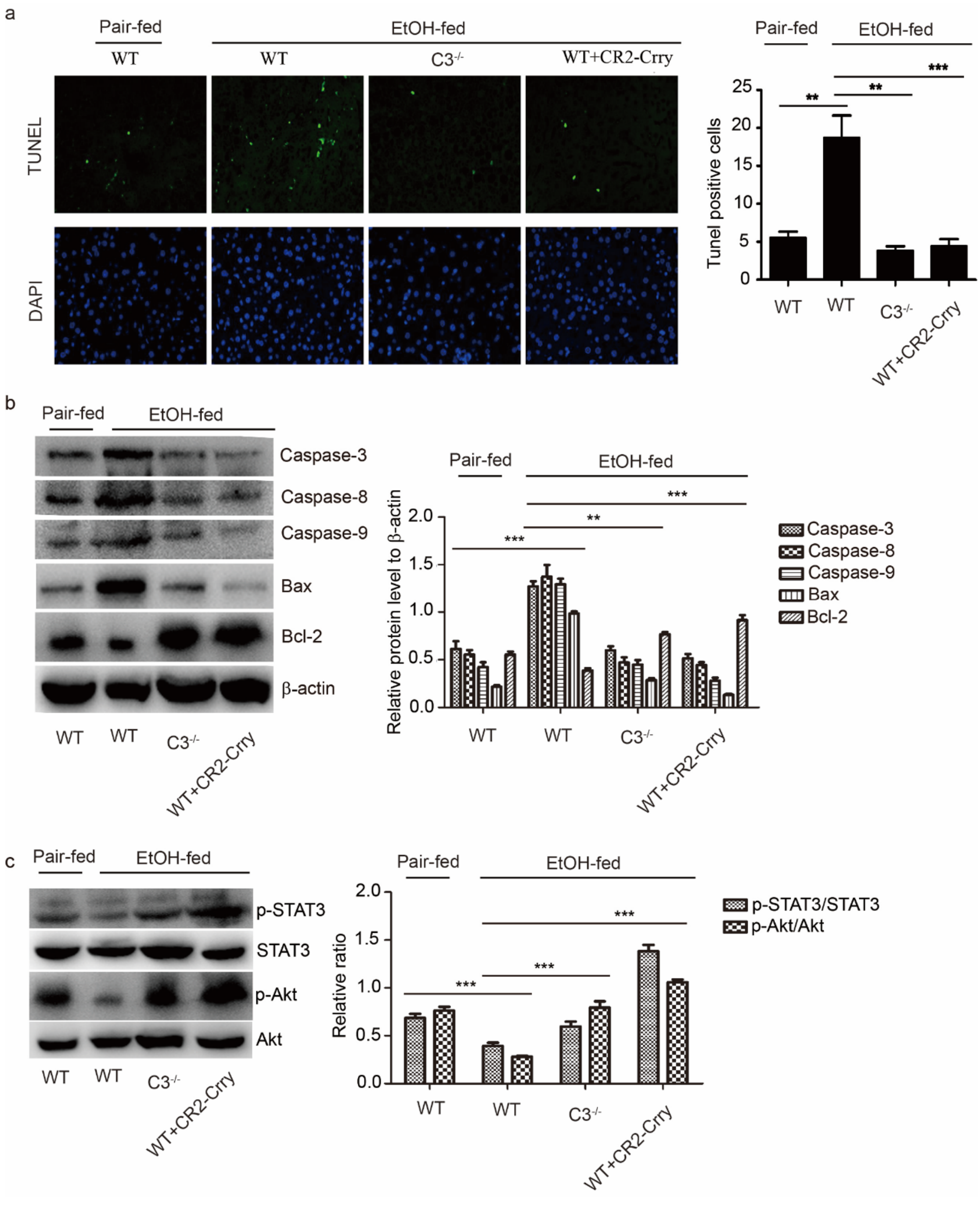

Fig. S2 Effect of CR2-Crry or C3 deficiency on the apoptosis in ethanol-fed mice. **a** Assessment of liver apoptosis by TUNEL. Green fluorescence represents an apoptosis positive signal (magnification, 40×). **b** Expression levels of CASP-3, CASP-8, CASP-

9, BCL2, and BAX in the liver were detected by western blot. **c** Expression levels of STAT3, p-STAT3, AKT, and p-AKT in the liver were examined by western blot. The data are representative of three independent experiments. The results are expressed as the mean ± SD. β-ACTIN was used as internal control. $^{**}P < 0.01$,$^{***}P < 0.001$.
