# Supplementary material for: Complement C3 activation regulates the production of tRNA-derived fragments Gly-tRFs and promotes alcohol-induced liver injury and steatosis
Source: Cell Res. 2019 May 10;29(7):548–61. doi: 10.1038/s41422-019-0175-2 (PMC6796853; doi:10.1038/s41422-019-0175-2)
Supplement: Supplementary file 3 — Supplementary information, Figure S3 [file 41422_2019_175_MOESM3_ESM.pdf]

Supplementary information, Fig. S3

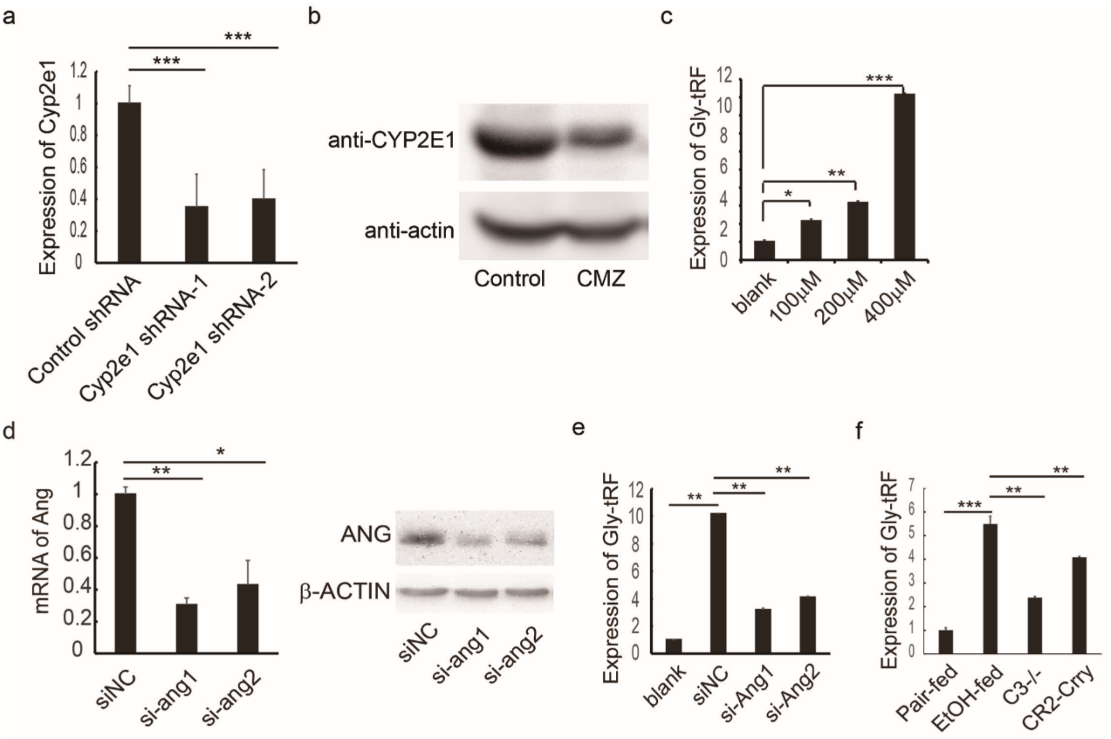

Fig. S3 C3 and oxidative stress were involved in regulation of Gly-tRF expression. **a** AAV9-shRNAs were delivered to the mice *via* tail vein injection. The knockdown effect of *Cyp2e1* shRNAs was evaluated by qRT-PCR. **b** The CMZ inhibitor was used to knock down *Cyp2e1*. The expresson of CYP2E1 was determined. **c** AML-12 cells were cultured with DMEM-F12 and treated with increasing concentrations of H_2O_2. Gly-tRF expression was detected by qRT-PCR. U6 was used as an internal control for RNA loading. **d** AML12 cells were transfected with *Ang* siRNA, and the effects of siRNA knockdown were assessed by qRT-PCR and western blot. **e** AML12 cells were transfected with *Ang* siRNA and treated with 300 μM H_2O_2 after 12 h. Gly-tRF expression was detected by qRT-PCR. NC, negative control. **f** Gly-tRF expression in pair-fed or ethanol-fed WT, *C3*$^{-/-}$ or CR2-Crry-treated mice. The data are representative of three independent experiments. The results are expressed as mean ± SD. **P* < 0.05, ***P* < 0.01, ****P* < 0.001.
