# Supplementary material for: Complement C3 activation regulates the production of tRNA-derived fragments Gly-tRFs and promotes alcohol-induced liver injury and steatosis
Source: Cell Res. 2019 May 10;29(7):548–61. doi: 10.1038/s41422-019-0175-2 (PMC6796853; doi:10.1038/s41422-019-0175-2)
Supplement: Supplementary file 4 — Supplementary information, Figure S4 [file 41422_2019_175_MOESM4_ESM.pdf]

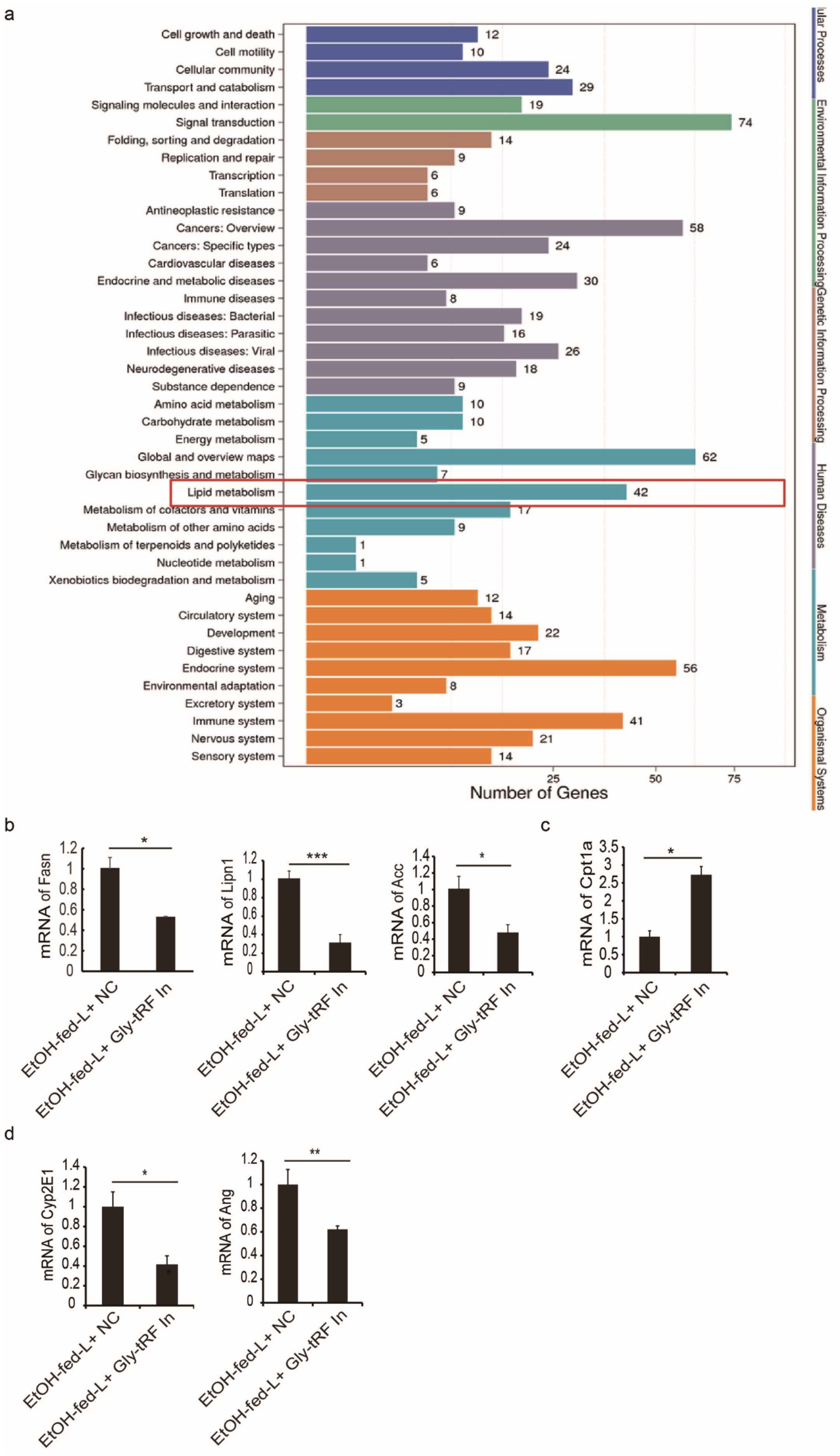

a
Cell growth and death
Cell motility
Cellular community
Transport and catabolism
Signaling molecules and interaction
Signal transduction
Folding, sorting and degradation
Replication and repair
Transcription
Translation
Antineoplastic resistance
Cancers: Overview
Cancers: Specific types
Cardiovascular diseases
Endocrine and metabolic diseases
Immune diseases
Infectious diseases: Bacterial
Infectious diseases: Parasitic
Infectious diseases: Viral
Neurodegenerative diseases
Substance dependence
Amino acid metabolism
Carbohydrate metabolism
Energy metabolism
Global and overview maps
Glycan biosynthesis and metabolism
Lipid metabolism
Metabolism of cofactors and vitamins
Metabolism of other amino acids
Metabolism of terpenoids and polyketides
Nucleotide metabolism
Xenobiotics biodegradation and metabolism
Aging
Circulatory system
Development
Digestive system
Endocrine system
Environmental adaptation
Excretory system
Immune system
Nervous system
Sensory system
12
10
24
29
19
74
14
9
6
6
9
58
24
6
30
8
19
16
26
18
9
10
10
5
62
7
42
17
9
1
1
5
12
14
22
17
56
8
3
41
21
14
25
50
75
Number of Genes
ular Processes
Environmental Information Processing
Genetic Information Processing
Human Diseases
Metabolism
Organismal Systems
b
mRNA of Fasn
mRNA of Lipn1
mRNA of Acc
c
mRNA of Cpt1a
d
mRNA of Cyp2E1
mRNA of Ang
*
***
**
EtOH-fed-L+ NC
EtOH-fed-L+ Gly-tRF In

Fig. S4 The Gly-tRF-associated pathways and -related genes. **a** Following treatment with Gly-tRF inhibitors, hepatic tissues were subjected to transcriptome profiling. Red arrow highlights Gly-tRF involvement in lipid metabolism pathway. **b** Hepatic expression of *Fasn*, *Lipin1*, *Acc.* **c** Hepatic expression of *Cpt1a*. **d** The expression of *Cyp2e1* and *Ang* was detected by qRT-PCR. The data are representative of three independent experiments. The results are expressed as mean ± SD. *P < 0.05, **P < 0.01, ***P < 0.001.
