# Supplementary material for: Complement C3 activation regulates the production of tRNA-derived fragments Gly-tRFs and promotes alcohol-induced liver injury and steatosis
Source: Cell Res. 2019 May 10;29(7):548–61. doi: 10.1038/s41422-019-0175-2 (PMC6796853; doi:10.1038/s41422-019-0175-2)
Supplement: Supplementary file 5 — Supplementary information, Figure S5 [file 41422_2019_175_MOESM5_ESM.pdf]

Supplementary information, Fig. S5

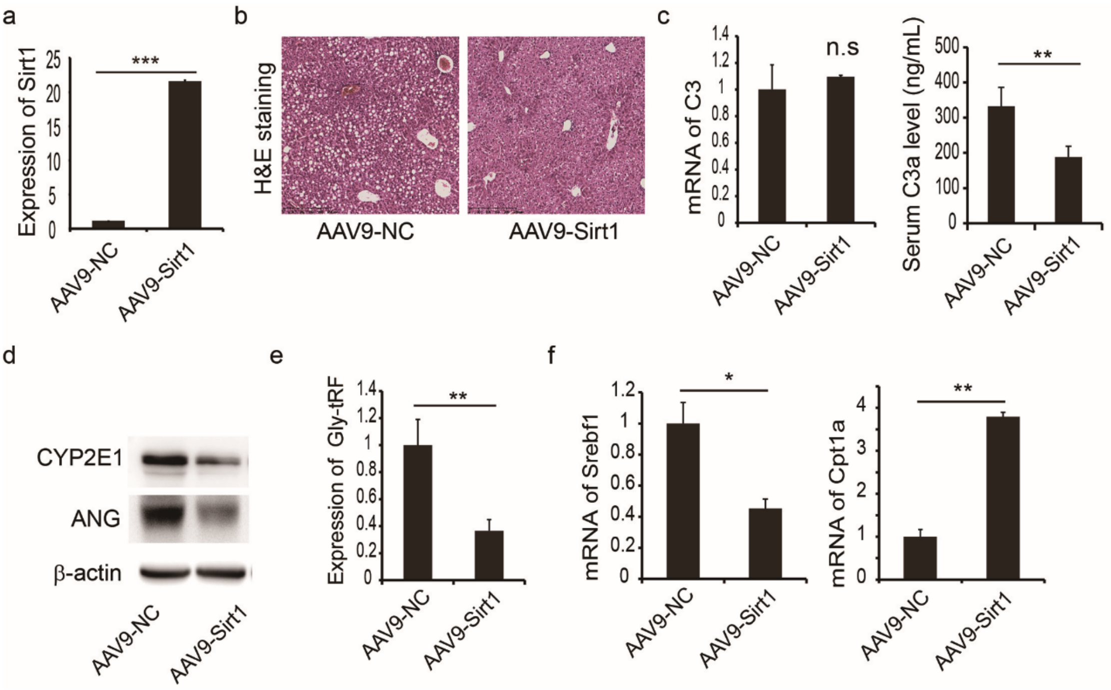

Fig. S5 Effect of overexpression of *Sirt1* on gene expression. **a** The expression of *Sirt1* was assessed. **b** H&E staining. **c** C3 mRNA and serum C3a level. **d, e** The effect of overexpression of *Sirt1* on the CYP2E1, ANG, and Gly-tRF expression. **f** The effect of overexpression of *Sirt1* on the *Srebp1* or *Cpt1a* expression. The data are representative of three independent experiments. The results are expressed as the mean ± SD. **P* < 0.05, ***P* < 0.01, ****P* < 0.001.
