# Supplementary material for: Complement C3 activation regulates the production of tRNA-derived fragments Gly-tRFs and promotes alcohol-induced liver injury and steatosis
Source: Cell Res. 2019 May 10;29(7):548–61. doi: 10.1038/s41422-019-0175-2 (PMC6796853; doi:10.1038/s41422-019-0175-2)
Supplement: Supplementary file 6 — Supplementary information, Figure S6 [file 41422_2019_175_MOESM6_ESM.pdf]

Supplementary information, Fig. S6

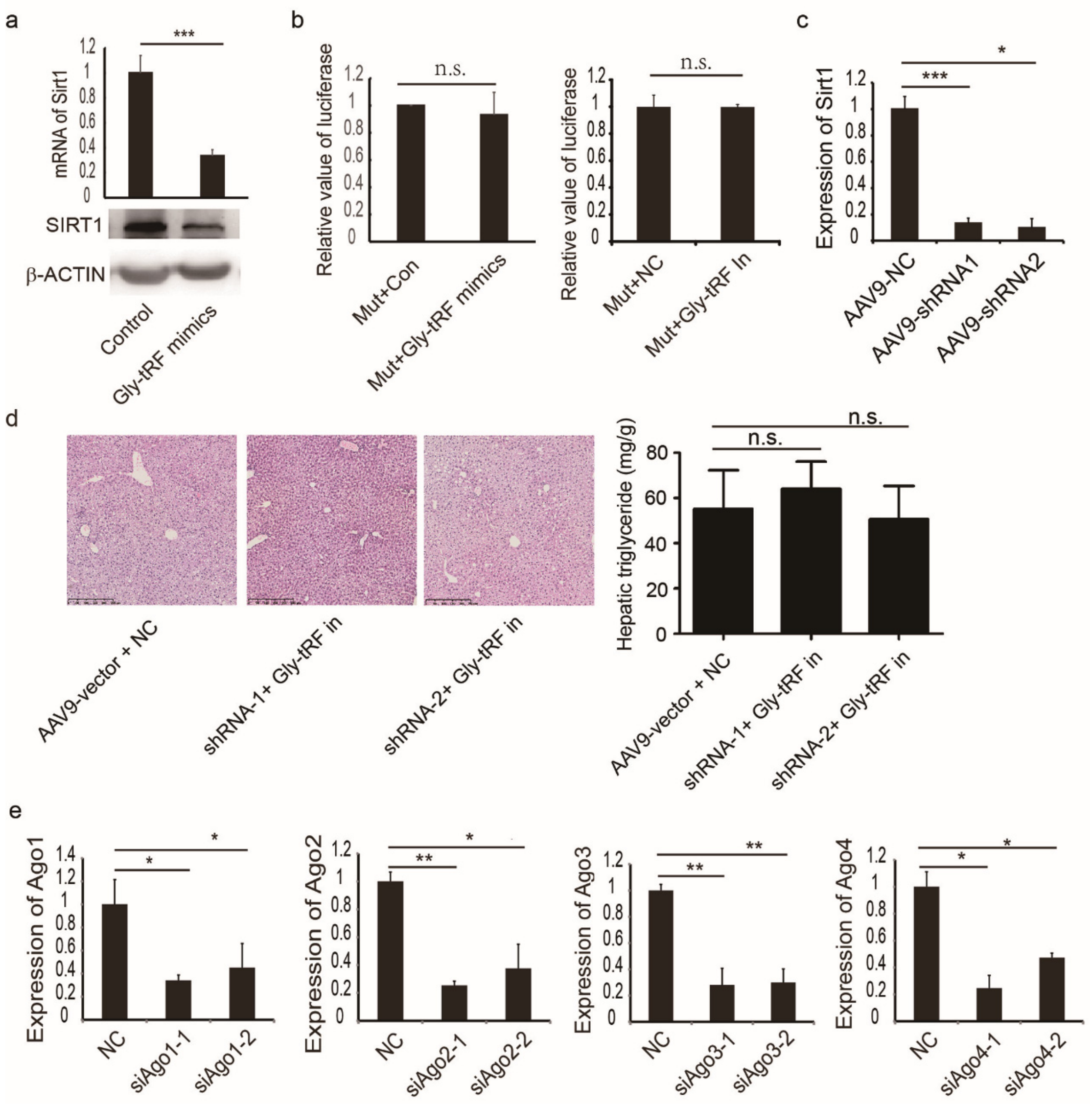

Fig. S6 Effect of Gly-tRF on *Sirt1* expression. **a** AML12 cells were transfected with Gly-tRF mimics or corresponding controls, and the expression of *Sirt1* was detected by qRT-PCR and western blot. Random sequence was used as control for Gly-tRF mimics. **b** Mutant was co-transfected with Gly-tRF mimics, Gly-tRF inhibitor, or corresponding controls, and after 48 h cells were collected for luciferase assays. n.s., not significant. **c** The knockdown effect of shRNAs of *Sirt1* was evaluated. **d** AAV9-shRNAs were used

to knock down *Sirt1* and then treated with Gly-tRF inhibitor (20 nm/mouse, i.p. twice per week). The effect of Gly-tRF inhibitor on steatosis was determined by H&E staining and hepatic triglycerides. **e** AML12 cells were transfected with NC or siRNAs to knock down *Ago1*, *Ago2*, *Ago3*, or *Ago4*, and the effect of siRNA knockdown was assessed by qRT-PCR. n.s., not significant. The data are representative of three independent experiments. The results are expressed as the mean ± SD. *$P < 0.05$, **$P < 0.01$, ***$P < 0.001$.
