# Supplementary material for: Complement C3 activation regulates the production of tRNA-derived fragments Gly-tRFs and promotes alcohol-induced liver injury and steatosis
Source: Cell Res. 2019 May 10;29(7):548–61. doi: 10.1038/s41422-019-0175-2 (PMC6796853; doi:10.1038/s41422-019-0175-2)
Supplement: Supplementary file 7 — Supplementary information, Table S1 [file 41422_2019_175_MOESM7_ESM.pdf]

Supplementary information, Table S1 Primers used in this study

gene	Forward primers	Reverse primers
ang	GCCTGTCCACTTCGATGAGT	GTTCGTTTTTCTGGCCCCTG
lipin1	CCCTCGATTTCAACGTACCC	GCAGCCTGTGGCAATTCA
ppara	AGAGCCCCATCTGTCCTCTC	ACTGGTAGTCTGCAAAACCAAA
fasn	GCCGTGTCCTTCTACCACAA	GGATCGGAGCATCTCTGGTG
srebp1	CGGGACAGCTTAGCCTCTAC	TCCATTGCTGGTACCGTGAG
sirt1	TTAATCAGGTAGTTCCTCGGTGCC	ACCACCTAGCCTATGACACAACTC
acc	TGAGGAGGACCGCATTTATC	GAAGCTTCCTTTGTGACCAG
Icam-1	ACGTGCTGTATGGTCCTCG	GTGGGGCTTGTCCCTTGA
Cxcl4	CGTGGACATCTACTCTTTC	CAGCCTGTAAACTGGGTA
Il-1β	AACCAACAAGTGATATTCTCCA	GGTGTGCCGTCTTTCATTAC
Ly6g	GAACCCTCACAGCCACCT	CTCAGCAAATGCCCAAAT
Cpt1a	GGAGGACCCTGAGGCATCTA	ACTGTAGCCTGGTGGGTTTG
Cyp2e1	GTCATCCCCAAGGGTACA	CAGAATAGCAGACAAAAGCA
sirt1-3'UTR	GGGGAGCTCCACTATTGAAGCTGTCCG GATTCAG	GGGAAGCTTGCAGGCTCTACCACAGTG ATAGG
mut1	AGCAGAGTGTGCAGAGTGGTCAAAACT TGGTGCTC	CTGCACACTCTGCTCTCTGGTTGTATTAC AAG
mut2	GATCAACGTAAGAAGGATACTTACTGC ATCGTT	CTTCTTACGTTGATCTTAATTTTTGAGTG CTCCAGAC
ago1	ACGCACCATGTACTTCGCTT	GGTCCAAGGGGGTCGGA
ago2	ACAGCCAGCATCGAACATGA	GGGACTCTCAGTGAGCAGCTTAT
ago3	TCACAAACATGGCGTCAGGA	ACAGCCAGCATCGAACATGA
ago4	CCAGCTCTGTCACACCTACG	TGGTAGGGACCACCTGAGAGA
gapdh	CCCACTAACATCAAATGGGG	CCTTCCACAATGCCAAAGTT
U6	CGCTTCGGCAGCACATATAC	GAACGCTTCACGAATTTGCGT
Gly-tRF	GTTCAGTGGTAGAATTCTCGCAA	
RTQ	CGAATTCTAGAGCTCGAGGCAGGCGAC ATGGCTGGCTAGTTAAGCTTGGTACCG AGCTCGGATCCACTAGTCCTTTTTTTTT TTTTTTTTTTTTTTVN (V is A, G, or C; N is A, G, C, or T)	
UniR	CGAATTCTAGAGCTCGAGGCAGG	
