# Supplementary material for: Complement C3 activation regulates the production of tRNA-derived fragments Gly-tRFs and promotes alcohol-induced liver injury and steatosis
Source: Cell Res. 2019 May 10;29(7):548–61. doi: 10.1038/s41422-019-0175-2 (PMC6796853; doi:10.1038/s41422-019-0175-2)
Supplement: Supplementary file 8 — Supplementary information, Table S2 [file 41422_2019_175_MOESM8_ESM.pdf]

Supplementary information, Table S2 Genes related to lipid metabolism pathway

Genes associated with lipid metabolism pathway. Fold change > 2 for analysis.

Symbol	Fold Change (tRF-Gly In/NC)	Up-Regulation (tRF-Gly In/NC)	Symbol	Fold Change (tRF-Gly In/NC)	Down-Regulation (tRF-Gly In/NC)
Hsd17b7	2.015250545	Up	Cyp2c55	0.131849315	Down
Acsl1	2.073909979	Up	Cyp2b10	0.140174297	Down
Mgll	2.145274875	Up	Lpin1	0.14204931	Down
Fads2	2.157726389	Up	Cyp2c29	0.180185126	Down
Ehhadh	2.17794853	Up	Cyp2c54	0.277006387	Down
Cpt1a	2.296079579	Up	Cyp2c50	0.400548118	Down
Cyp4a14	2.353182687	Up	Acnat2	0.405277402	Down
Cyp4a10	2.385528036	Up	Aacs	0.418573351	Down
Pla2g6	2.402658327	Up	Lpl	0.428571429	Down
Cyp4a32	2.598130841	Up	Akr1b7	0.438566553	Down
Agpat9	2.621993127	Up	Alg12	0.444047619	Down
Fads3	2.673267327	Up	Osbpl8	0.450980392	Down
Crat	2.679731243	Up	Lpcat4	0.454545455	Down
Elovl3	2.694581281	Up	Cyp3a11	0.461630826	Down
Apoa4	2.699387142	Up	Akr1b10	0.480263158	Down
Ppara	2.745752783	Up	Ppargc1a	0.483221477	Down
Acot2	2.859210526	Up	Eif2ak3	0.484162896	Down
Plin2	2.974021948	Up	Sult1d1	0.496101764	Down
Cyp2c69	3.030614576	Up			
Acot3	3.107423581	Up			
Cyp3a41b	4.576923077	Up			
Acot1	5.903073286	Up			
Cyp3a16	11.56589147	Up			
Sirt1	2.416666667	Up			
Cidec	14.80555556	Up			
